# Association between frailty and postoperative delirium after transcatheter aortic valve replacement: a meta-analysis

**DOI:** 10.3389/fpsyt.2026.1840158

**Published:** 2026-05-21

**Authors:** Zhiheng Liu, Ning Wu, Fan Wu, Tao Liu, Kai Ren

**Affiliations:** 1 Department of Cardiovascular Surgery, Air Force 986 Hospital, Air Force Medical University, Xi’an, China; 2 Department of Cardiovascular Surgery, Xijing Hospital, Air Force Medical University, Xi’an, China

**Keywords:** aortic stenosis, frailty, meta-analysis, postoperative delirium, transcatheter aortic valve replacement

## Abstract

**Background:**

Postoperative delirium (POD) is a common complication following transcatheter aortic valve replacement (TAVR) and is associated with adverse outcomes in older patients. Frailty, a multidimensional geriatric syndrome, has been increasingly recognized as a potential risk factor for POD. However, existing evidence remains inconsistent. This meta-analysis aimed to evaluate the association between frailty and POD after TAVR.

**Methods:**

A systematic search of PubMed, Embase, and Web of Science was conducted from inception to January 22, 2026. Cohort studies evaluating the association between preprocedural frailty and POD after TAVR were included. Odds ratios (ORs) with 95% confidence intervals (CIs) were pooled using a random-effects model accounting for the influence of potential heterogeneity.

**Results:**

Ten cohort studies involving 7,702 patients were included. Frailty was present in 2,062 (26.8%) patients, and 786 (10.2%) developed POD. Pooled analysis showed that frailty was significantly associated with an increased risk of POD after TAVR (OR: 2.17, 95% CI: 1.60–2.95; I^2^ = 55%). The association was stronger in studies with sample size ≥ 500 compared with < 500 (OR: 2.74 vs. 1.38; *p* for subgroup difference < 0.001). The effect estimates were consistent across subgroups stratified by study design, age, sex, frailty assessment methods, follow-up duration, analytic models, and study quality (all *p* for subgroup difference > 0.05). Notably, studies using CAM-ICU to diagnose POD showed a stronger association than those using DSM criteria or other methods (OR: 3.60 vs. 1.56 and 2.53; *p* = 0.006). Meta-regression identified sample size as a significant source of heterogeneity (*p* = 0.02).

**Conclusions:**

Frailty is associated with an increased risk of POD after TAVR. These findings highlight the importance of frailty assessment for perioperative risk stratification and support targeted strategies to prevent delirium in high-risk patients undergoing TAVR.

**Systematic review registration:**

https://www.crd.york.ac.uk/prospero/, identifier CRD420261352173.

## Introduction

Postoperative delirium (POD) is a frequent and clinically significant complication following transcatheter aortic valve replacement (TAVR), particularly in older patients with severe aortic stenosis who often present with multiple comorbidities and reduced physiological reserve (1–3). The reported incidence of POD after TAVR varies widely, generally ranging from approximately 5% to 25%, depending on patient characteristics and diagnostic methods (1). POD has been consistently associated with adverse outcomes, including prolonged hospitalization, increased healthcare costs, functional decline, institutionalization, and higher short- and long-term mortality (4, 5). Several risk factors for POD after TAVR have been identified, including advanced age, cognitive impairment, comorbidities, non-transfemoral access, and the use of general anesthesia (GA) (1, 6). However, these factors do not fully explain the variability in POD risk. Given the growing use of TAVR in elderly populations, identifying robust and clinically applicable predictors of POD is essential to improve perioperative risk stratification and guide preventive strategies.

Frailty, a multidimensional geriatric syndrome characterized by diminished physiological reserve and increased vulnerability to stressors (7), has emerged as a key determinant of outcomes in patients undergoing TAVR (8, 9). Frailty can be assessed using a variety of validated instruments, including multidimensional indices and physical performance–based measures (10, 11). Previous studies have demonstrated that frailty is associated with increased risks of mortality, complications, and poor functional recovery after TAVR (12, 13). Importantly, frailty may also predispose patients to POD through mechanisms such as impaired neurocognitive reserve, chronic inflammation, and reduced resilience to perioperative stress (14, 15). Although several observational studies have explored the association between frailty and POD after TAVR, their findings have been inconsistent, likely due to differences in study design, frailty assessment methods, and definitions of POD (16–25). To date, a comprehensive synthesis of the available evidence is lacking. Therefore, this meta-analysis was conducted to systematically evaluate the association between frailty and POD in patients undergoing TAVR and to explore potential sources of heterogeneity across studies.

## Methods

The meta-analysis was carried out in accordance with established methodological guidance, following the principles outlined in the PRISMA 2020 statement (26) and the Cochrane Handbook for Systematic Reviews and Meta-Analyses (27), encompassing protocol planning, study selection, data collection, statistical analysis, and results interpretation. The study protocol was registered prospectively in the PROSPERO database (registration number: CRD420261352173).

### Database search

A systematic literature search was conducted in PubMed, Embase, and Web of Science to identify studies that met the eligibility criteria for inclusion. The search strategy was constructed using the combination of the following terms: (1) “frailty” OR “frail”; (2) “transcatheter aortic valve implantation” OR “TAVI” OR “transcatheter aortic valve replacement” OR “TAVR”; (3) “confusion” OR “delirium” OR “acute encephalopathy” OR “cognitive dysfunction” OR “cognitive impairment” OR “cognitive disorder” OR “altered mental status” OR “organic brain syndrome” OR “acute encephalopathy”. Only full-text, peer-reviewed articles published in English and involving human participants were eligible for inclusion. Additionally, the reference lists of relevant reviews and original studies were manually examined to identify further potentially eligible publications. All databases were searched from their inception up to January 22, 2026. Detailed search strategies for each database are presented in **Supplementary File 1**.

### Study inclusion and exclusion criteria

The selection of studies was guided by the PICOS principle:

P (Population): Adult patients (≥ 18 years) with severe AS and related indications undergoing TAVR, regardless of access route, valve type, or anesthesia strategy.

I (Exposure): Preoperative frailty assessed prior to TAVR using validated multidimensional frailty instruments (e.g., Clinical Frailty Scale [CFS], frailty index [FI], Fried phenotype) or validated physical performance–based measures reflecting frailty domains (e.g., Short Physical Performance Battery [SPPB] or gait speed). Frailty had to be reported as a categorical variable (frail vs. non-frail) based on predefined cutoffs according to those in the original studies.

C (Comparator): Patients classified as non-frail before TAVR within each study.

Outcome (O): POD occurring after TAVR, defined using validated diagnostic criteria or tools (e.g., Confusion Assessment Method [CAM], Diagnostic and Statistical Manual of Mental Disorders [DSM] criteria, or clinician-diagnosed delirium), with sufficient data to estimate effect sizes.

S (Study design): Observational studies (prospective or retrospective cohort, nested case–control studies) with longitudinal follow-up, or *post-hoc* analysis of randomized controlled trials.

Studies were excluded if they met any of the following criteria: (1) did not include patients undergoing TAVR or did not report TAVR-specific results separately; (2) did not assess frailty prior to the procedure or lacked a clear and reproducible definition of frailty; (3) reported frailty only as a continuous variable without providing categorical comparisons or sufficient data for conversion; (4) did not report POD or provided insufficient data to estimate the association between frailty and POD; (5) were review articles, editorials, case reports, or meta-analyses; or (6) included overlapping populations. To minimize the risk of including overlapping populations, we compared study characteristics such as study center, geographic location, recruitment period, and patient population. When potential overlap was suspected, only the study with the largest sample size was included.

### Study quality assessment

Two reviewers independently performed the literature search, screened studies for eligibility, extracted data, and assessed study quality. Any disagreements were resolved through discussion, and when necessary, a third investigator was consulted to reach consensus. The methodological quality of the included studies was evaluated using the Newcastle–Ottawa Scale (NOS) (28), which assesses study quality across three domains: selection, comparability, and outcome assessment. NOS scores range from 1 to 9, with studies scoring ≥ 8 considered to be of high quality.

### Data collection

Data extraction was conducted independently by two reviewers using a standardized and pre-tested data collection form. Extracted variables included study characteristics (first author, publication year, study design, and country), patient characteristics (diagnosis, sample size, mean age, sex distribution, access route for TAVR, and type of anesthesia), exposure details (timing and methods for the evaluation of frailty, and number of patients with frailty in each study), follow-up duration, methods for the diagnosis of POD, number of patients who developed POD, and variables adjusted for in multivariable analyses examining the association between frailty and POD after TAVR.

### Statistical analyses

The association between frailty and POD in patients after TAVR was evaluated by pooling odds ratios (ORs) with their corresponding 95% confidence intervals (CIs) (27). When available, the most fully adjusted effect estimates were preferentially extracted; otherwise, unadjusted estimates were used when adjusted data were not reported. When adjusted estimates were not reported, crude data were used to calculate unadjusted ORs and 95% CIs. No conversions from other effect measures (e.g., risk ratios or hazard ratios) were required. When necessary, effect estimates and their standard errors were derived from the reported 95% CIs or *p* values. Before pooling, all effect measures were converted to the natural logarithmic scale to improve normality and ensure variance stabilization for meta-analysis (27). To evaluate variability across studies, we applied the Cochrane Q test and calculated the I^2^ statistic (29). Heterogeneity was categorized based on I^2^ values as low (< 25%), moderate (25–75%), or high (> 75%). Pooled effect estimates were calculated using the inverse variance (IV) approach within a random-effects framework (DerSimonian–Laird method) to account for potential between-study variability (27). In addition, a sensitivity analysis was performed using a more conservative random-effects approach (Hartung–Knapp adjustment with restricted maximum likelihood [REML] estimation) to evaluate the robustness of the pooled estimates (27). Moreover, a sensitivity analysis restricted solely to the studies with most fully adjusted estimates was also performed. Sensitivity analyses were performed using a leave-one-out approach, in which each study was sequentially excluded to assess the stability and robustness of the pooled results (30). To identify possible sources of between-study variability, we performed predefined subgroup analyses stratified by study design (prospective vs. retrospective), sample size of the study, mean age of the patients, proportion of men, methods for evaluating frailty, follow-up duration, methods for the diagnosis of POD, analytic model (univariate vs. multivariate), and NOS scores. Median values of continuous variables were used as cutoffs to define subgroups, ensuring a balanced distribution of studies across subgroups, unless otherwise specified. Additionally, univariate meta-regression analyses were conducted to examine the potential impact of study-level characteristics on the association between frailty and POD after TAVR (27). The variables assessed included sample size, mean age, proportion of male participants, proportion of patients with transfemoral access, proportion of patients who received GA, and study quality scores via NOS. Publication bias was assessed through visual inspection of funnel plot symmetry and further evaluated using Egger’s regression test (31). A two-sided *p* value < 0.05 was considered indicative of statistical significance. All analyses were performed using RevMan (version 5.3; Cochrane Collaboration, Oxford, UK) and Stata (version 17.0; StataCorp, College Station, TX, USA).

## Results

### Database search results

**Figure 1:**
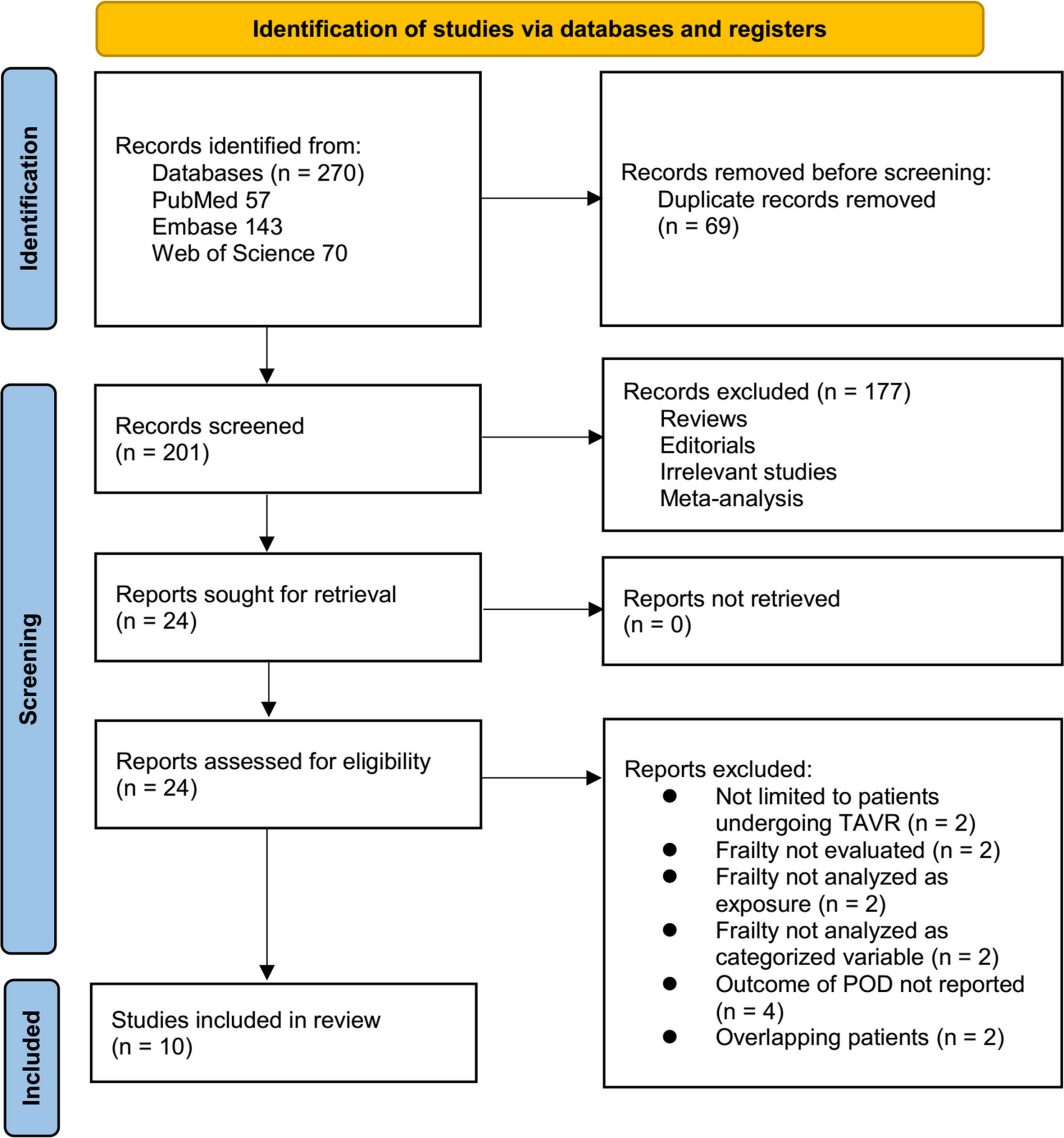
Flow diagram of the study selection process.

The study selection process is illustrated in **Figure 1**. A total of 270 records were retrieved from the three databases, of which 69 duplicates were removed. Following title and abstract screening, 177 records were excluded for not meeting the predefined inclusion criteria. The full texts of 24 articles were subsequently evaluated independently by two reviewers, and 14 were excluded for the reasons detailed in **Figure 1**. Ultimately, ten studies met the eligibility criteria and were included in the quantitative meta-analysis (16–25).

### Overview of study characteristics

The main characteristics of the included studies are summarized in **Table 1**. A total of 10 cohort studies, including seven prospective (16–21, 23) and three retrospective studies (22, 24, 25), published between 2016 and 2026 were included in this meta-analysis, comprising 7,702 patients undergoing TAVR for severe AS and related indications. The studies were conducted across multiple regions, including Europe (the Netherlands, Germany, and Poland), North America (Canada and the USA), Asia (Japan), and Australia, indicating broad geographic diversity. The mean age of patients ranged from 76.3 to 84.5 years, and the proportion of men ranged from 35.8% to 62.0%, reflecting an elderly population with a relatively balanced sex distribution. TF access was the predominant approach in most studies, although some cohorts included alternative access routes such as subclavian (16), axillary (21), or transapical approaches (17, 21, 25). The proportion of patients receiving GA varied substantially across studies from 43.0% to 100.0% in studies with the reported data. Frailty was assessed prior to the procedure using a variety of instruments, including multidimensional tools such as FI (16, 17, 24), Edmonton Frail Scale (EFS) (19, 23), Essential Frailty Toolset (EFT) (20), Groningen Frailty Indicator (GFI) (22), and Kihon Checklist (25), as well as domain-based measures such as impaired mobility or composite functional deficit (18, 21). Overall, 2,062 (26.8%) patients had frailty before TAVR. POD was assessed during hospitalization or within a short postoperative period using validated diagnostic criteria such as DSM-IV (16, 19, 21) or DSM-V (22, 23), and CAM-ICU (20, 25), or alternative methods such as the Intensive Care Delirium Screening Checklist (ICDSC) (18), chart-based delirium identification instrument (CHART-DEL) (17), or clinical diagnosis as evidenced by International Classification of Diseases (ICD) codes (24). Patients were followed for POD occurrence either within 2–7 days after TAVR (17, 19, 20, 23) or throughout hospitalization (16, 18, 21, 22, 24, 25). Accordingly, 786 (10.2%) patients developed POD after TAVR. Seven studies (16, 18–22, 24) performed multivariable analyses adjusting for key confounders, including age, sex, comorbidities, cognitive function, procedural characteristics, and anesthesia type, although the extent of adjustment differed across studies. Three studies only reported data from univariate studies (17, 23, 25).

**Table 1:** Characteristics of the included studies.

Study	Design	Country	Diagnosis	No. of patients included	Mean age (years)	Men (%)	Access route	GA (%)	Timing of frailty evaluation	Methods for frailty evaluation	No. of patients with frailty	Follow-up duration (days)	Definition of POD	No. of patients with POD	Variables adjusted
Assmann 2016 (16)	PC	The Netherlands	Severe symptomatic AS	89	80.4	43.0	Left SCA 100%	100.0	Before procedure	FI	44	During hospitalization	DSM-IV criteria	25	Age, PH, MMSE, IADL, and TUG
Bagienski 2017 (17)	PC	Poland	Severe symptomatic AS	141	82.0	36.9	TF 80.1%, TA 17.7%, others 2.2%	69.5	Before procedure	FI	55	4	CHART-DEL instrument (validated chart-based method)	29	None
Khan 2019 (18)	PC	Canada	Severe AS	234	82.2	59.4	NR	61.1	Before procedure	At least one deficit in ADL, TUG, and HGS	23	During hospitalization	ICDSC score	23	Age, GA, cognitive deficits, and depressive symptoms
Goudzwaard 2020 (19)	PC	The Netherlands	Severe symptomatic AS	543	79.1	55.0	TF 91%, others 9%	43.0	Before procedure	EFS	97	4	DSM-IV criteria	75	Age, prior stroke, renal dysfunction, limitation of mobility (gait speed), GA, non-TF access, and procedural time
Mauri 2021 (20)	PC	Germany	Severe native AS	661	82.3	48.7	TF 97.4%, other 2.6%	56.4	Before procedure	EFT	199	7	CAM-ICU	66	Age, male sex, AF, pneumonia, stroke, vascular complication, and GA
van der Wulp 2021 (21)	PC	The Netherlands	Severe symptomatic AS	511	80.0	44.8	TF 21.1%, axillary 71.4%, TA 7.4%	100.0	Before procedure	Impaired mobility (gait speed ≤ 0.83 m/s OR TUG ≥ 20 seconds)	180	During hospitalization	DSM-IV criteria	66	Age, BMI, previous delirium, and aortic valve area < 0.75 cm^2^
Dautzenberg 2022 (22)	RC	The Netherlands	Severe symptomatic AS	431	80.8	44.0	NR	NR	Before procedure	GFI	155	During hospitalization	DSM-V criteria	22	Age and sex
Ghezzi 2023 (23)	PC	Australia	Severe AS	32	82.5	62.0	TF 90%, subclavian 10%	NR	Before procedure	EFS	5	2	DSM-V criteria	7	None
Badwan 2025 (24)	RC	USA	Patients undergoing TAVR	4074	76.3	53.4	NR	NR	Before procedure	FI	836	During hospitalization	ICD codes	439	Age, sex, race, comorbidities, medications, BMI, and GFR
Kobayashi 2026 (25)	RC	Japan	Severe symptomatic AS	986	84.5	35.8	TF 95.8%, subclavian 2.6%, TA 1.6%	NR	Before procedure	KCL	468	During hospitalization	CAM-ICU	34	None

NR, not reported; PC, prospective cohort; RC, retrospective cohort; AS, aortic stenosis; TAVR, transcatheter aortic valve replacement; TF, transfemoral; TA, transapical; SCA, subclavian artery; GA, general anesthesia; FI, frailty index; EFS, Edmonton Frail Scale; EFT, Essential Frailty Toolset; GFI, Groningen Frailty Indicator; KCL, Kihon Checklist; ADL, activities of daily living; IADL, instrumental activities of daily living; TUG, Timed Up and Go test; HGS, handgrip strength; BMI, body mass index; GFR, glomerular filtration rate; POD, postoperative delirium; DSM, Diagnostic and Statistical Manual of Mental Disorders; CAM-ICU, Confusion Assessment Method for the Intensive Care Unit; ICDSC, Intensive Care Delirium Screening Checklist; CHART-DEL, chart-based delirium identification instrument; ICD, International Classification of Diseases; AF, atrial fibrillation; PH, pulmonary hypertension.

### Study quality evaluation

The methodological quality of the included studies was assessed using the NOS, with detailed results presented in **Table 2**. The NOS scores ranged from 6 to 9, indicating overall moderate to high methodological quality. Four studies (16, 18, 20, 21) achieved the maximum score of 9, reflecting strong cohort representativeness, appropriate selection of comparison groups, adequate exposure ascertainment, comprehensive adjustment for confounders, and reliable outcome assessment. Two studies scored 8, mainly due to minor limitations such as incomplete adjustment for additional confounders (22) or insufficient follow-up duration (19). The remaining four studies scored 6–7 (17, 23–25), primarily owing to limited control for confounding factors, lack of adjustment for key variables, or less rigorous outcome assessment methods. Notably, most studies adequately defined both frailty and POD and ensured that outcomes were not present at baseline (16–23, 25). Overall, the included studies were considered to be of moderate to high quality, supporting the reliability of the pooled estimates while acknowledging some heterogeneity in study design and methodological rigor.

**Table 2:** Study quality evaluation via the Newcastle-Ottawa Scale.

Study	Representativeness of the exposed cohort	Selection of the non-exposed cohort	Ascertainment of exposure	Outcome not present at baseline	Control for age	Control for other confounding factors	Assessment of outcome	Enough long follow-up duration	Adequacy of follow-up of cohort	Total
Assmann 2016 (16)	1	1	1	1	1	1	1	1	1	9
Bagienski 2017 (17)	1	1	1	1	0	0	1	0	1	6
Khan 2019 (18)	1	1	1	1	1	1	1	1	1	9
Goudzwaard 2020 (19)	1	1	1	1	1	1	1	0	1	8
Mauri 2021 (20)	1	1	1	1	1	1	1	1	1	9
van der Wulp 2021 (21)	1	1	1	1	1	1	1	1	1	9
Dautzenberg 2022 (22)	1	1	1	1	1	0	1	1	1	8
Ghezzi 2023 (23)	1	1	1	1	0	0	1	0	1	6
Badwan 2025 (24)	0	1	1	1	1	1	0	1	1	7
Kobayashi 2026 (25)	1	1	1	1	0	0	1	1	1	7

### Results of the meta-analysis

**Figure 2:**
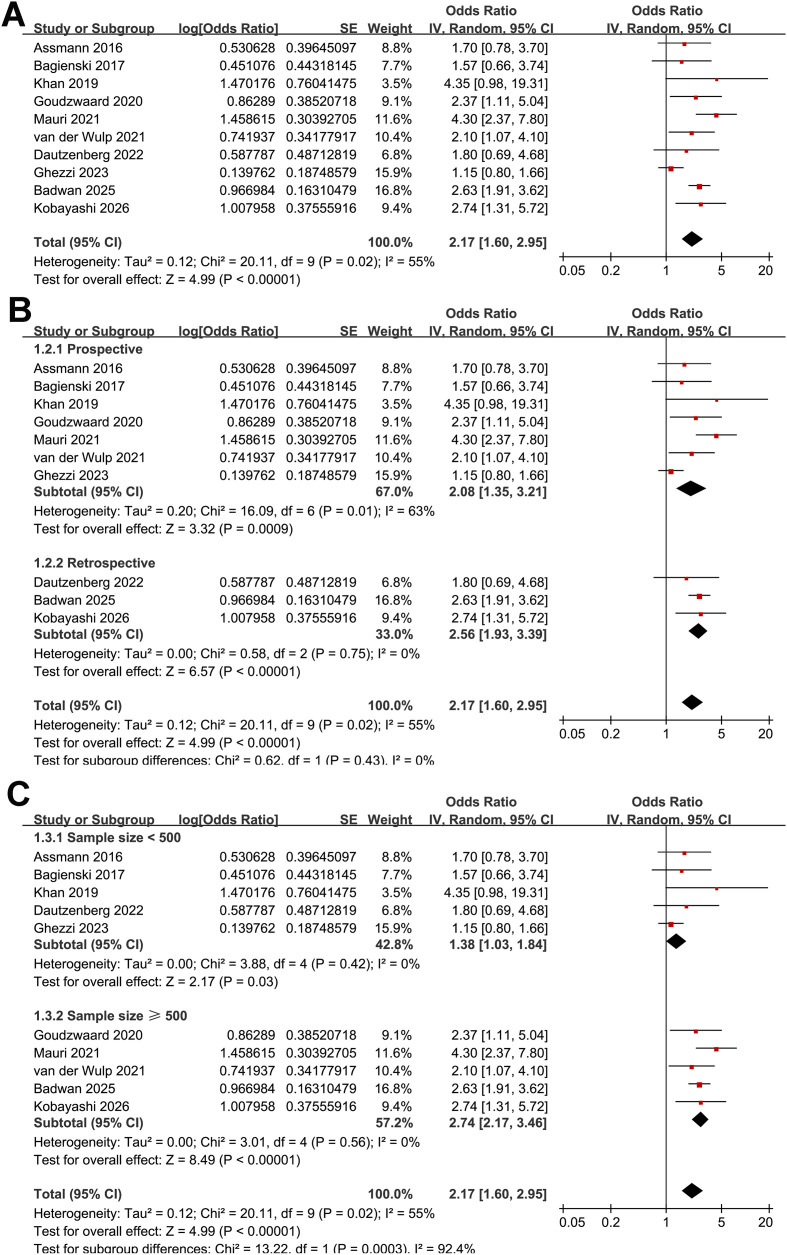
Forest plots showing the meta-analysis of the association between frailty and POD after TAVR: **(A)** forest plots for the overall meta-analysis; **(B)** forest plots for the subgroup analysis according to study design; and **(C)** forest plots for the subgroup analysis according to sample size.

The pooled analysis of the 10 studies (16–25) demonstrated that frailty prior to TAVR was associated with an increased incidence of POD (OR: 2.17, 95% CI: 1.60 to 2.94, *p* < 0.001; **Figure 2A**) with moderate heterogeneity (Cochrane Q test *p* = 0.02; I^2^ = 55%). Sensitivity analysis using the Hartung-Knapp adjustment with REML estimation yielded similar results (OR: 2.17, 95% CI: 1.60 to 2.95, *p* < 0.001; I^2^ = 53%; **Supplementary Figure 1**), supporting the robustness of the primary findings. Moreover, sensitivity analysis restricted solely to the studies with most fully adjusted estimates (16, 18–22, 24) also showed consistent results (OR: 2.60, 95% CI: 2.08 to 3.26, *p* < 0.001; I^2^ = 0%; **Supplementary Figure 2**). Leave-one-out sensitivity analyses yielded consistent results, with pooled ORs ranging from 1.97 to 2.54 (all *p* < 0.05), indicating the robustness of the overall estimate.

### Results of the subgroup analysis

**Figure 3:**
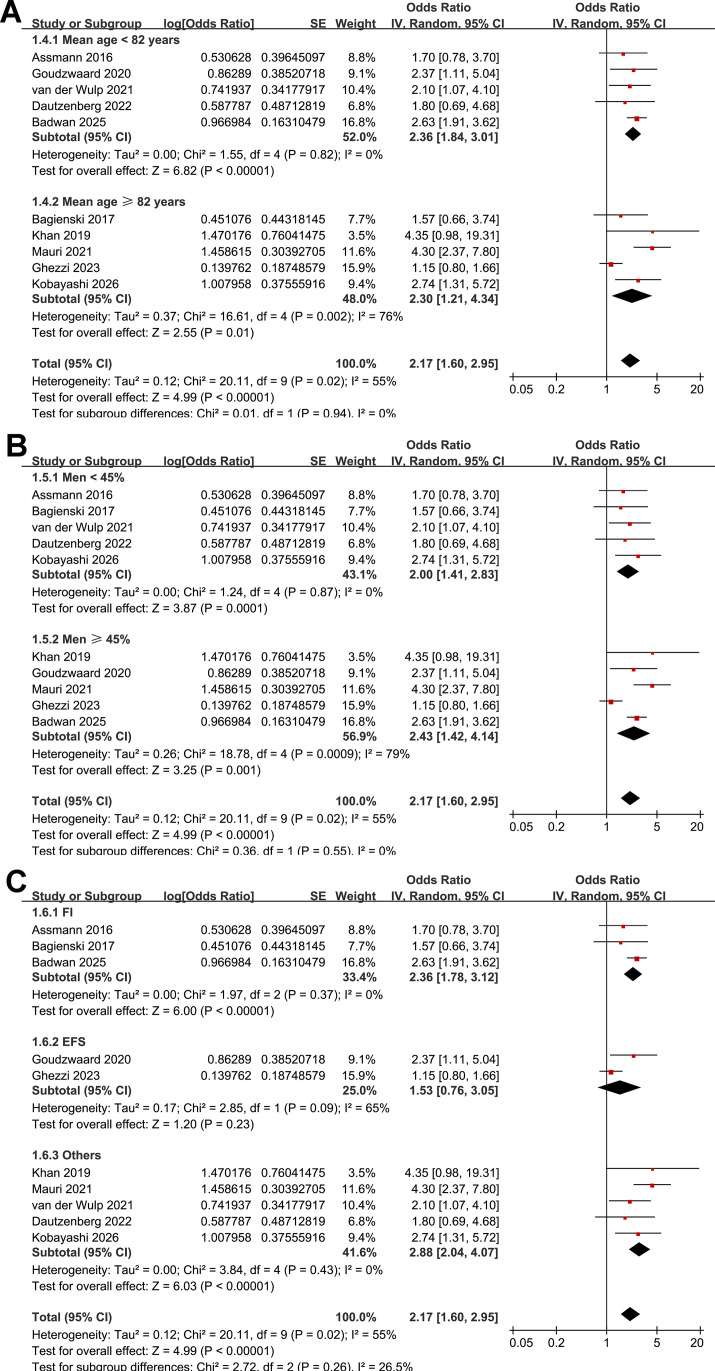
Forest plots showing the subgroup analysis of the association between frailty and POD after TAVR: **(A)** forest plots for the subgroup analysis according to the mean age of the patients; **(B)** forest plots for the subgroup analysis according to the proportion of men; and **(C)** forest plots for the subgroup analysis according to the methods for evaluating frailty.

**Figure 4:**
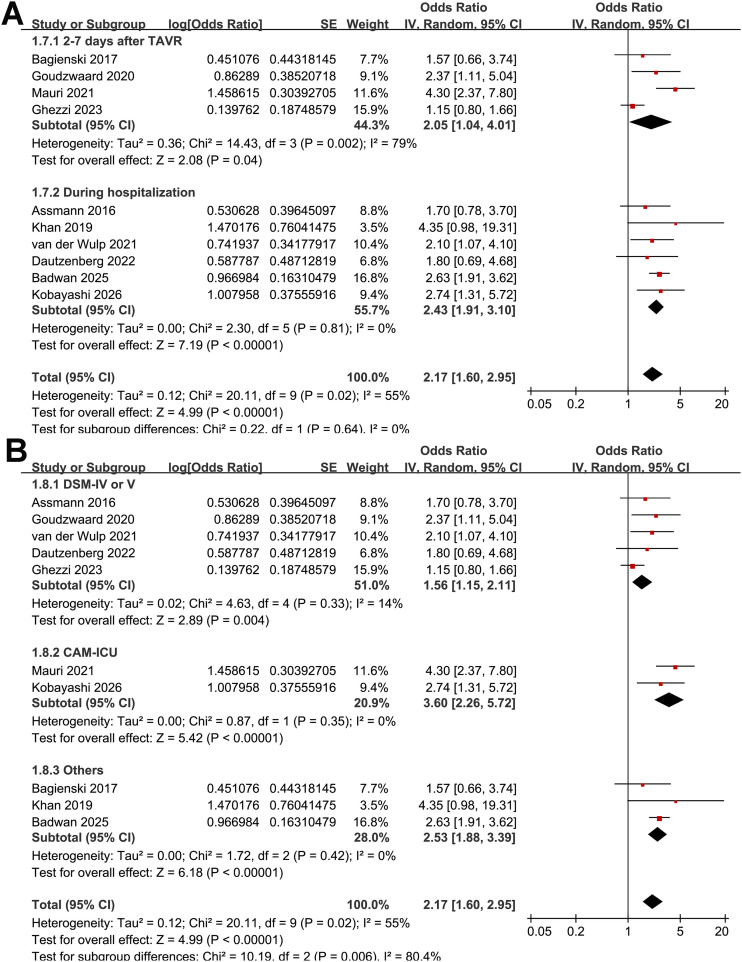
Forest plots showing the subgroup analysis of the association between frailty and POD after TAVR: **(A)** forest plots for the subgroup analysis according to the follow-up duration; and **(B)** forest plots for the subgroup analysis according to the methods for the diagnosis of POD.

**Figure 5:**
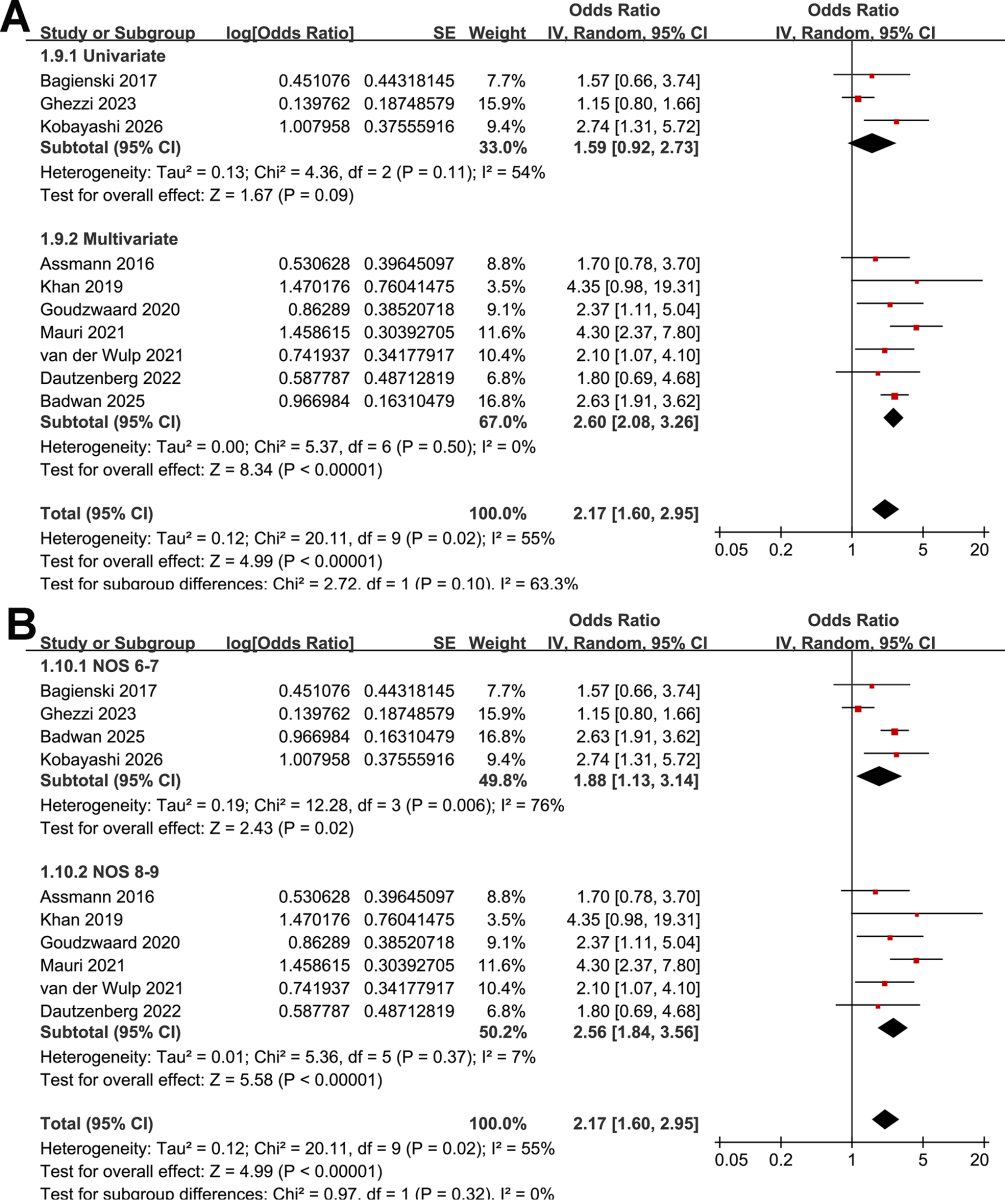
Forest plots showing the subgroup analysis of the association between frailty and POD after TAVR: **(A)** forest plots for the subgroup analysis according to the analytic models; and **(B)** forest plots for the subgroup analysis according to the study quality score in NOS.

Subgroup analyses showed similar results in prospective and retrospective studies (OR: 2.08 vs. 2.56, *p* for subgroup difference = 0.43; **Figure 2B**). However, a stronger association between frailty and POD after TAVR was observed in studies with a sample size ≥ 500 compared with those with < 500 participants (OR: 2.74 vs. 1.38; *p* for subgroup difference < 0.001; **Figure 2C**). Notably, stratification by sample size appeared to reduce heterogeneity, with low heterogeneity observed within each subgroup (both I^2^ = 0%). However, this finding should be interpreted cautiously given the limited number of studies included in each subgroup. In addition, similar results were observed for studies with the mean ages of the patients < 82 and ≥ 82 years (OR: 2.36 vs. 2.30, *p* for subgroup difference = 0.94; **Figure 3A**) and between studies with the proportion of men < 45% and ≥ 45% (OR: 2.00 vs. 2.43, *p* for subgroup difference = 0.55; **Figure 3B**). Further subgroup analyses showed that the association between frailty and POD did not differ significantly across studies using different frailty assessment methods (FI, EFS, or other measures; OR: 2.36, 1.53, and 2.88, respectively; *p* for subgroup difference = 0.26; **Figure 3C**), nor between studies with POD assessed at 2–7 days after TAVR versus during hospitalization (OR: 2.05 vs. 2.43, *p* for subgroup difference = 0.64; **Figure 4A**). However, the estimate for the EFS subgroup should be interpreted with caution, as it was based on only two studies (19, 23) with limited sample size, substantial heterogeneity (I^2^ = 65%), and a wide CI crossing unity. Interestingly, subsequent analyses indicated that the method used to diagnose POD may influence the results, with a stronger association between frailty and POD observed in studies using CAM-ICU compared with those using chart-based screening tools or ICD codes, and those using DSM-IV/V criteria (OR: 3.60, 2.53, and 1.56, respectively; *p* for subgroup difference = 0.006; **Figure 4B**). No significant differences were observed between studies reporting univariate versus multivariable analyses (OR: 1.59 vs. 2.60; *p* for subgroup difference = 0.10; **Figure 5A**), or between studies with NOS scores of 6–7 and those with scores of 8–9 (OR: 1.88 vs. 2.56; *p* for subgroup difference = 0.32; **Figure 5B**).

### Results of the meta-regression analysis

Univariate meta-regression analysis was performed to explore potential sources of heterogeneity, as summarized in **Table 3**. The results suggested that sample size was associated with the effect estimate (coefficient = 0.00010, *p* = 0.02) and may partly contribute to between-study heterogeneity. However, this finding should be interpreted with caution due to the limited number of studies and the inherent constraints of study-level meta-regression. Other variables, inlcuding mean age, proportion of men, TF access rate, and GA rate were not significantly associated with the effect size (all *p* > 0.05), suggesting that these factors did not materially influence the pooled results. Study quality, as assessed by the NOS, also showed no statistically significant association with the effect estimate (*p* = 0.10), although it explained a moderate proportion of heterogeneity (adjusted R^2^ = 42.1%). Overall, these findings indicate that variation in sample size may be a key contributor to between-study heterogeneity, while other study-level characteristics appear to have limited impact.

**Table 3:** Results of univariate meta-regression analysis.

Variables	OR for the association between frailty and POD after TAVR			
	Coefficient	95% CI	*p* values	Adjusted R^2^
Sample size	0.00010	0.00004 to 0.00016	0.02	100%
Mean age (years)	-0.019	-0.169 to 0.132	0.78	0%
Men (%)	-0.0099	-0.0513 to 0.0315	0.60	0%
TF (%)	0.0027	-0.0091 to 0.0145	0.61	0%
GA (%)	0.0025	-0.0120 to 0.0170	0.70	0%
NOS	0.21	-0.05 to 0.47	0.10	42.1%

OR, odds ratio; CI, confidence interval; POD, postoperative delirium; TAVR, transcatheter aortic valve replacement; TF, transfemoral; GA, general anesthesia; NOS, Newcastle–Ottawa Scale.

### Publication bias

**Figure 6:**
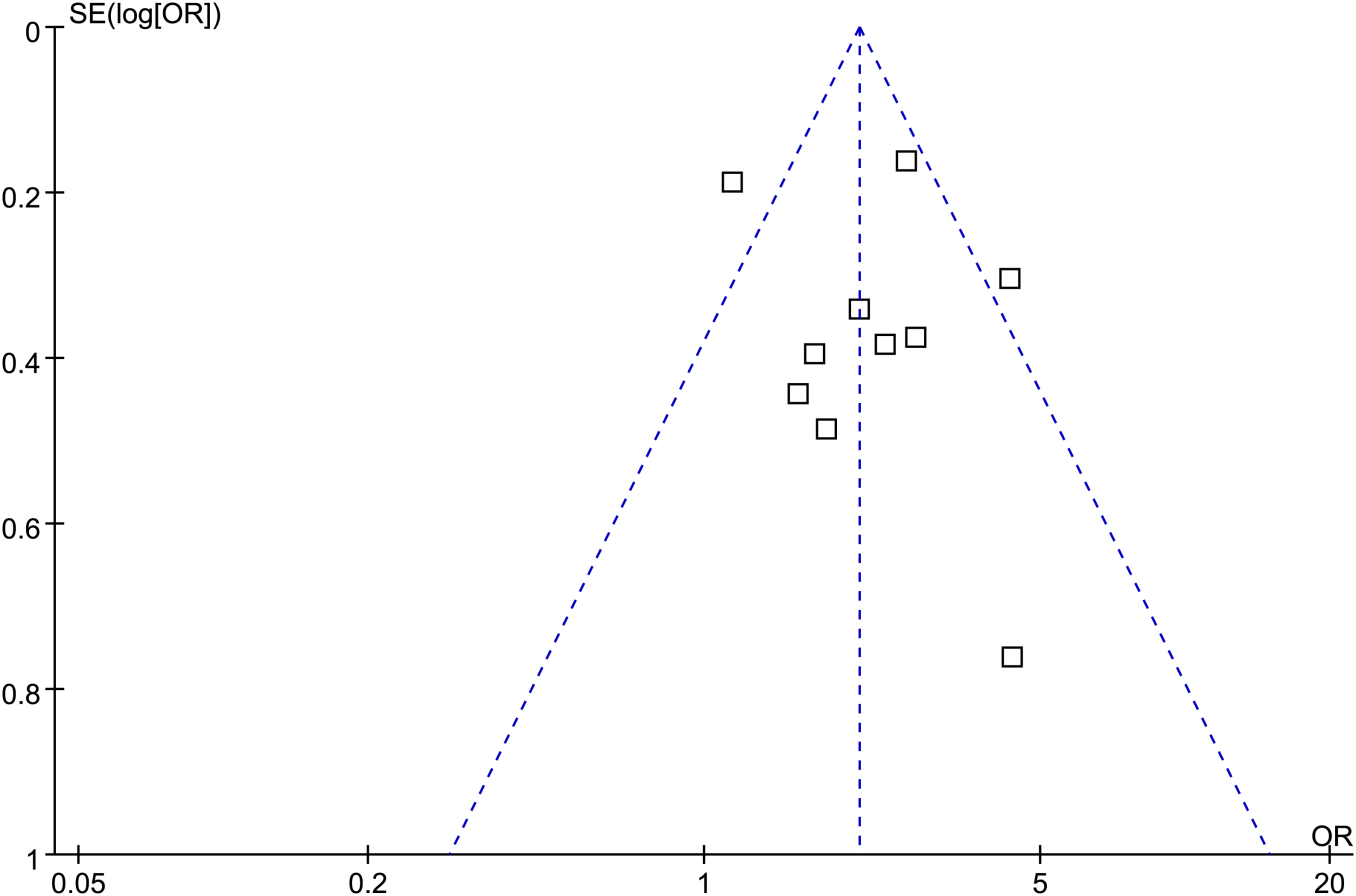
Funnel plots evaluating the publication bias for the meta-analysis of the association between frailty and POD after TAVR.

As illustrated in **Figure 6**, the funnel plots assessing the association between frailty and POD in patients after TAVR appeared generally symmetrical. In line with this observation, Egger’s regression test did not detect significant publication bias (*p* = 0.66). Nevertheless, these findings should be interpreted with caution due to the relatively small number of included studies (k = 10).

## Discussion

This meta-analysis synthesized evidence from 10 cohort studies involving 7,702 patients undergoing TAVR and found that preprocedural frailty is associated with an increased risk of POD. The association was generally consistent across subgroup and sensitivity analyses, supporting the overall robustness of the findings. Moderate heterogeneity was observed, and although subgroup and meta-regression analyses suggested that study-level characteristics such as sample size may contribute to variability, these findings should be interpreted cautiously given the limited number of studies. Overall, these results suggest that frailty may serve as a clinically relevant marker for identifying patients at higher risk of POD, supporting its consideration in perioperative risk stratification for TAVR candidates.

The incidence of POD observed in this meta-analysis (10.2%) is lower than that typically reported after conventional open cardiac surgery, where rates in elderly populations often range from 20% to 50% (32, 33). This difference likely reflects the less invasive nature of TAVR, which avoids cardiopulmonary bypass and is associated with reduced systemic inflammation and physiological stress (34). In this context, the impact of frailty on postoperative complications may be attenuated compared with open surgical procedures. Supporting this hypothesis, a recent meta-analysis by Dorey et al. (35) reported that frailty was associated with an increased risk of postoperative atrial fibrillation in patients undergoing conventional cardiac surgery, whereas no significant association was observed in TAVR populations. Despite this potential attenuation, our findings demonstrate that frailty remains a significant predictor of POD even in the TAVR setting. This suggests that the vulnerability captured by frailty—particularly reduced neurocognitive reserve and diminished resilience to stressors—persists even with less invasive procedures. Therefore, while TAVR may reduce the overall incidence of POD, frailty continues to identify a subgroup of patients at increased risk.

Several pathophysiological and clinical mechanisms may explain the observed association between frailty and POD after TAVR. Frailty reflects a state of reduced physiological and neurocognitive reserve, rendering patients more susceptible to perioperative stressors such as anesthesia, hemodynamic fluctuations, inflammation, and microembolic cerebral injury (36–38). Chronic systemic inflammation, neuroinflammation, and impaired blood–brain barrier integrity in frail individuals may further predispose to delirium (39, 40). In addition, frailty is often accompanied by cognitive impairment, malnutrition, reduced mobility, and polypharmacy, all of which are established risk factors for POD (41, 42). Clinically, frail patients may have diminished capacity to compensate for acute insults during and after TAVR, leading to a higher likelihood of acute brain dysfunction (43). These mechanisms are consistent with current conceptual models of delirium as the result of an interaction between baseline vulnerability and precipitating factors.

The subgroup analyses provide additional insight into the robustness and potential modifiers of this association. A stronger association was observed in larger studies (sample size ≥ 500), which may reflect greater statistical precision compared with smaller studies that are more susceptible to random error. Notably, stratification by sample size was accompanied by a reduction in heterogeneity within subgroups. However, this observation should be interpreted cautiously, as the number of included studies was limited, and subgroup analyses are inherently exploratory. Therefore, while sample size may contribute to between-study variability, it cannot be considered a definitive explanation for the observed heterogeneity. In contrast, the association between frailty and POD was consistent across subgroups defined by age, sex, frailty assessment methods, follow-up duration, analytic models, and study quality, indicating that the relationship is relatively stable across different study conditions. However, the method used for POD diagnosis significantly influenced the effect estimates, with stronger associations observed in studies using CAM-ICU compared with DSM-based or other methods. This may reflect differences in sensitivity and detection frequency, as CAM-ICU allows for systematic and repeated bedside assessment, potentially capturing more delirium events, whereas DSM-based diagnoses or administrative coding may underestimate incidence (44). The findings of the meta-regression analysis further support the results of the subgroup analyses. Sample size was identified as a significant moderator, explaining all between-study heterogeneity, whereas other study-level characteristics, including age, sex distribution, access route, anesthesia type, and study quality, were not significantly associated with the effect size. These results suggest that the observed association is relatively consistent across these study-level characteristics. However, residual confounding cannot be excluded. Together, the subgroup and meta-regression analyses provide additional context for interpreting the main findings and sources of heterogeneity, although these results should be considered exploratory.

In addition, GA is a well-recognized perioperative factor that may contribute to the development of POD through mechanisms such as altered cerebral perfusion, neuroinflammation, and exposure to sedative agents (45). In the present analysis, although GA rate varied substantially across studies, meta-regression did not identify it as a significant moderator of the association between frailty and POD after TAVR. This finding should be interpreted cautiously. Given that frail patients may be more vulnerable to the neurocognitive effects of anesthesia (46), a potential interaction between frailty and GA cannot be excluded. Moreover, study-level meta-regression may not adequately reflect individual-level associations, particularly when adjustment for confounders differs across studies.

This study has several strengths. First, it included an up-to-date and comprehensive literature search across major databases, ensuring that the most recent evidence was incorporated. Second, all included studies were cohort designs, enhancing the relevance of the findings to real-world clinical practice. Third, multiple sensitivity, subgroup, and meta-regression analyses were conducted to explore heterogeneity and assess the robustness of the results. Fourth, the study systematically evaluated different methods for frailty and POD assessment, providing additional insights into potential sources of variability.

Several limitations should also be acknowledged. First, there was substantial heterogeneity in the definitions and assessment methods of both frailty and POD across the included studies. Frailty was evaluated using a variety of multidimensional instruments as well as domain-based measures such as impaired mobility, which may not fully capture the complexity of the frailty construct. Similarly, POD was identified using different approaches, including prospective bedside tools (e.g., CAM-ICU), DSM-based criteria, and administrative or chart-based methods, which are not methodologically equivalent and may differ in sensitivity and specificity. Although subgroup analyses according to frailty assessment methods and POD diagnostic approaches were performed, residual heterogeneity cannot be excluded. Second, the lack of individual patient data precluded detailed evaluation of the influence of demographic, clinical, and procedural characteristics on the association. Third, subgroup and meta-regression analyses were based on a limited number of studies and should be interpreted cautiously. These analyses are inherently underpowered at the study level and may yield unstable or spurious associations, particularly when the number of included studies is small. For example, the subgroup analysis according to frailty assessment methods should be interpreted cautiously. In particular, the EFS subgroup included only two studies (19, 23) with heterogeneous designs and differing methodological quality, resulting in imprecise estimates and limiting the reliability of comparisons across frailty instruments. In addition, subgroup analysis according to GA exposure could not be performed because four included studies did not report GA rates, and available data were insufficient for reliable stratification. Furthermore, the assessment of GA as a potential effect modifier was limited to study-level meta-regression, which may be underpowered and susceptible to ecological bias when the number of studies is small. Third, although most studies adjusted for key confounders, residual confounding cannot be excluded, particularly for factors such as baseline cognitive function, inflammatory status, and perioperative management (47). In addition, although most included studies reported multivariable-adjusted estimates, some studies contributed only unadjusted data, and the combination of these estimates may limit causal inference despite consistent findings across subgroup analyses. Fourth, the observational nature of the included studies limits the ability to infer causality, and the results should be interpreted as associations rather than causal relationships. Besides, only English-language publications were included, which may introduce language bias and increase the risk of selective reporting, as relevant studies published in other languages may have been missed. Fifth, although no significant publication bias was detected, the relatively small number of studies may limit the power of formal tests, and the possibility of selective reporting cannot be entirely excluded. Moreover, although the methodological quality of included studies was assessed using the NOS, this tool has inherent limitations and may not comprehensively address key sources of bias, such as residual confounding, heterogeneity in frailty definitions, and differences in outcome ascertainment. Therefore, the overall risk of bias may be underestimated, and the findings should be interpreted cautiously. Finally, variations in perioperative care protocols, including anesthesia strategies and delirium prevention measures, may also contribute to heterogeneity.

From a clinical perspective, these findings support the routine assessment of frailty in patients undergoing TAVR as part of comprehensive risk stratification. Identifying frail patients may facilitate targeted interventions to mitigate the risk of POD, such as optimization of comorbidities, medication review, early mobilization, and implementation of non-pharmacological delirium prevention strategies. However, evidence regarding the effectiveness of structured geriatric interventions remains limited. For example, a recent quasi-experimental study evaluating preoperative comprehensive geriatric assessment (CGA) in older TAVR patients did not demonstrate a significant reduction in POD incidence, although it highlighted the complexity of delirium prevention and the need for integrated perioperative care models (48). This suggests that frailty assessment may be a useful component of perioperative risk stratification, although its role in guiding specific management strategies remains to be established. Future research should focus on prospective studies with standardized definitions of frailty and POD to improve comparability across studies. Studies incorporating individual patient data may allow for more precise adjustment of confounders and better exploration of effect modifiers. In addition, well-designed interventional studies are needed to determine whether integrating frailty assessment into perioperative care pathways can improve clinical outcomes in this high-risk population.

## Conclusions

In conclusion, this meta-analysis demonstrates that frailty is associated with an increased risk of POD in patients undergoing TAVR. These findings underscore the importance of frailty as a key determinant of perioperative vulnerability and support its incorporation into routine clinical assessment. While the results provide important insights for risk stratification, further research is needed to establish effective interventions to mitigate delirium risk in frail TAVR patients.

## Data Availability

The original contributions presented in the study are included in the article/**Supplementary Material**. Further inquiries can be directed to the corresponding author.

## References

[1] Ma X Chu H Han K Shao Q Yu Y Jia S . Postoperative delirium after transcatheter aortic valve replacement: An updated systematic review and meta-analysis. J Am Geriatr Soc. (2023) 71:646–60. doi: 10.1111/jgs.18104. PMID: 36419366

[2] Mangold AS Benincasa S Sanders BM Patel K Mitrev L . Neurological complications after transcatheter aortic valve replacement: A review. Anesth Analg. (2024) 139:986–96. doi: 10.1213/ane.0000000000007087. PMID: 39136954

[3] Padilla-Serrano A Pozo JIG Lorente EC Cruz AC . Cardiovascular and non-cardiovascular complications of transcatheter aortic valve implantation. Catheter Cardiovasc Interv. (2025) 106:1461–74. doi: 10.1002/ccd.31702. PMID: 40546053

[4] Prasitlumkum N Mekritthikrai R Kewcharoen J Kanitsoraphan C Mao MA Cheungpasitporn W . Delirium is associated with higher mortality in transcatheter aortic valve replacement: Systemic review and meta-analysis. Cardiovasc Interv Ther. (2020) 35:168–76. doi: 10.1007/s12928-019-00592-y. PMID: 31154617

[5] Melegari G Villa M Regazzi M Cassina T . Delirium after transcatheter aortic valve replacement: Incidence, predictive factors, and 1-year functional and cognitive outcomes. J Cardiothorac Vasc Anesth. (2026). doi: 10.1053/j.jvca.2026.01.037. PMID: 41765733

[6] Ochani S Adnan A Siddiqui A Kalwar A Kukreja S Ahmad M . Postoperative delirium in 47 379 individuals undergoing transcatheter aortic valve replacement: A systematic review and meta-analysis. Ann Med Surg (Lond). (2023) 85:4476–90. doi: 10.1097/01.ccm.0000457580.70349.65. PMID: 37663694 PMC10473306

[7] Kalladi R Elavally S Rojan PM Usharani EN . Frailty: An overview. J Family Med Prim Care. (2026) 15:39–44. doi: 10.4103/jfmpc.jfmpc_1748_25. PMID: 41816172 PMC12975101

[8] van Mourik MS Velu JF Lanting VR Limpens J Bouma BJ Piek JJ . Preoperative frailty parameters as predictors for outcomes after transcatheter aortic valve implantation: A systematic review and meta-analysis. Neth Heart J. (2020) 28:280–92. doi: 10.1016/j.jacc.2019.08.828. PMID: 32189208 PMC7190780

[9] Anand A Harley C Visvanathan A Shah ASV Cowell J MacLullich A . The relationship between preoperative frailty and outcomes following transcatheter aortic valve implantation: A systematic review and meta-analysis. Eur Heart J Qual Care Clin Outcomes. (2017) 3:123–32. doi: 10.1093/ehjqcco/qcw030. PMID: 28927173 PMC5862025

[10] Cappe M Laterre PF Dechamps M . Preoperative frailty screening, assessment and management. Curr Opin Anaesthesiol. (2023) 36:83–8. doi: 10.1097/aco.0000000000001221. PMID: 36476726 PMC9794163

[11] Deng Y Sato N . Global frailty screening tools: Review and application of frailty screening tools from 2001 to 2023. Intractable Rare Dis Res. (2024) 13:1–11. doi: 10.5582/irdr.2023.01113. PMID: 38404737 PMC10883846

[12] Kempton H Hall R Hungerford SL Hayward CS Muller DWM . Frailty and transcatheter valve intervention: A narrative review. Catheter Cardiovasc Interv. (2024) 104:155–66. doi: 10.1002/ccd.31076. PMID: 38819861

[13] Niebauer J Bäck C Bischoff-Ferrari HA Dehbi HM Szekely A Völler H . Preinterventional frailty assessment in patients scheduled for cardiac surgery or transcatheter aortic valve implantation: A consensus statement of the European Association for Cardio-Thoracic Surgery (EACTS) and the European Association of Preventive Cardiology (EAPC) of the European Society of Cardiology (ESC). Eur J Prev Cardiol. (2024) 31:146–81. doi: 10.1093/ejcts/ezad181. PMID: 37804173

[14] Schindele D McDonough J Müller-Wolff T . Impact of frailty on postoperative delirium in ICU patients aged 65 and older: A systematic review. BMJ Open. (2026) 16:e108249. doi: 10.1136/bmjopen-2025-108249. PMID: 41571420 PMC12829360

[15] Sung TY Oh CS . Frailty assessment in perioperative geriatric patients: A narrative review. Anesth Pain Med (Seoul). (2026) 21:51–66. doi: 10.17085/apm.25480. PMID: 41667249 PMC12890551

[16] Assmann P Kievit P van der Wulp K Verkroost M Noyez L Bor H . Frailty is associated with delirium and mortality after transcatheter aortic valve implantation. Open Heart. (2016) 3:e000478. doi: 10.1136/openhrt-2016-000478. PMID: 28008356 PMC5174802

[17] Bagienski M Kleczynski P Dziewierz A Rzeszutko L Sorysz D Trebacz J . Incidence of postoperative delirium and its impact on outcomes after transcatheter aortic valve implantation. Am J Cardiol. (2017) 120:1187–92. doi: 10.1016/j.amjcard.2017.06.068. PMID: 28826892

[18] Khan MM Lanctôt KL Fremes SE Wijeysundera HC Radhakrishnan S Gallagher D . The value of screening for cognition, depression, and frailty in patients referred for TAVI. Clin Interv Aging. (2019) 14:841–8. doi: 10.2147/cia.s201615. PMID: 31190770 PMC6512610

[19] Goudzwaard JA de Ronde-Tillmans M de Jager TAJ Lenzen MJ Nuis RJ van Mieghem NM . Incidence, determinants and consequences of delirium in older patients after transcatheter aortic valve implantation. Age Ageing. (2020) 49:389–94. doi: 10.1093/ageing/afaa001. PMID: 32091096 PMC7577406

[20] Mauri V Reuter K Körber MI Wienemann H Lee S Eghbalzadeh K . Incidence, risk factors and impact on long-term outcome of postoperative delirium after transcatheter aortic valve replacement. Front Cardiovasc Med. (2021) 8:645724. doi: 10.3389/fcvm.2021.645724. PMID: 33842564 PMC8032857

[21] van der Wulp K van Wely MH Schoon Y Vart P Olde Rikkert MGM Morshuis WJ . Geriatric assessment in the prediction of delirium and long-term survival after transcatheter aortic valve implantation. J Thorac Cardiovasc Surg. (2021) 161:2095–102:e3. doi: 10.1016/j.jtcvs.2020.02.076. PMID: 32241615

[22] Dautzenberg L van Aarle TTM Stella PR Emmelot-Vonk M Weterman MA Koek HL . The impact of frailty on adverse outcomes after transcatheter aortic valve replacement in older adults: A retrospective cohort study. Catheter Cardiovasc Interv. (2022) 100:439–48. doi: 10.1002/ccd.30320. PMID: 35830708 PMC9545405

[23] Ghezzi ES Psaltis PJ Loetscher T Davis D Boord MS Greaves D . Factors associated with cognitive decline and delirium after transcatheter aortic valve implantation: Preliminary evidence. Delirium. (2023). doi: 10.56392/001c.74542. Published online May 12, 2023.

[24] Badwan O Motairek I Zghyer F Puri R Reed G Krishnaswamy A . Frailty predicts geriatric and cardiovascular adverse outcomes after TAVR: A 5-year real-world analysis. JACC Adv. (2025) 4:102326. doi: 10.1016/j.jacadv.2025.102326. PMID: 41263744 PMC12805150

[25] Kobayashi D Saitoh M Hori K Tajima S Takizawa K Sakamoto J . Multidimensional frailty and mortality in transcatheter aortic valve implantation patients - Prognostic value of the Kihon checklist. Circ Rep. (2026) 8:153–61. doi: 10.1253/circrep.cr-25-0221. PMID: 41523943 PMC12782907

[26] Page MJ Moher D Bossuyt PM Boutron I Hoffmann TC Mulrow CD . PRISMA 2020 explanation and elaboration: Updated guidance and exemplars for reporting systematic reviews. BMJ. (2021) 372:n160. doi: 10.31222/osf.io/gwdhk 33781993 PMC8005925

[27] Higgins J Thomas J Chandler J Cumpston M Li T Page M . Cochrane Handbook for Systematic Reviews of Interventions version 6.2. In: The Cochrane Collaboration (2021). Available online at: https://www.training.cochrane.org/handbook.

[28] Wells GA Shea B O’Connell D Peterson J Welch V Losos M . The Newcastle-Ottawa Scale (NOS) for assessing the quality of nonrandomised studies in meta-analyses (2010). Available online at: http://www.ohri.ca/programs/clinical_epidemiology/oxford.asp (Accessed February 25, 2026).

[29] Higgins JP Thompson SG . Quantifying heterogeneity in a meta-analysis. Stat Med. (2002) 21:1539–58. doi: 10.1191/026921699666884611. PMID: 12111919

[30] Marušić MF Fidahić M Cepeha CM Farcaş LG Tseke A Puljak L . Methodological tools and sensitivity analysis for assessing quality or risk of bias used in systematic reviews published in the high-impact anesthesiology journals. BMC Med Res Methodol. (2020) 20:121. 32423382 10.1186/s12874-020-00966-4 PMC7236513

[31] Egger M Davey Smith G Schneider M Minder C . Bias in meta-analysis detected by a simple, graphical test. BMJ. (1997) 315:629–34. doi: 10.1136/bmj.315.7109.629. PMID: 9310563 PMC2127453

[32] Yan E Veitch M Saripella A Alhamdah Y Butris N Tang-Wai DF . Association between postoperative delirium and adverse outcomes in older surgical patients: A systematic review and meta-analysis. J Clin Anesth. (2023) 90:111221. doi: 10.1016/j.jclinane.2023.111221. PMID: 37515876

[33] Igwe EO Nealon J O’Shaughnessy P Bowden A Chang HR Ho MH . Incidence of postoperative delirium in older adults undergoing surgical procedures: A systematic literature review and meta-analysis. Worldviews Evid Based Nurs. (2023) 20:220–37. doi: 10.1111/wvn.12649. PMID: 37128953

[34] Spears J Al-Saiegh Y Goldberg D Manthey S Goldberg S . TAVR: A review of current practices and considerations in low-risk patients. J Interv Cardiol. (2020) 2020:2582938. doi: 10.1155/2020/2582938. PMID: 33447165 PMC7781688

[35] Dorey TW Bohne LJ Fatehi Hassanabad A Duttchen K Wilton SB Chun R . The relationship between frailty and postoperative atrial fibrillation in patients undergoing cardiac surgery: A systematic review and meta-analysis. J Cardiac Surg. (2025) 2025:1336346. doi: 10.1016/j.cjca.2024.08.230. PMID: 38826717

[36] Lin HS McBride RL Hubbard RE . Frailty and anesthesia - Risks during and post-surgery. Local Reg Anesth. (2018) 11:61–73. doi: 10.2147/lra.s142996. PMID: 30323657 PMC6178933

[37] Jin Z Rismany J Gidicsin C Bergese SD . Frailty: The perioperative and anesthesia challenges of an emerging pandemic. J Anesth. (2023) 37:624–40. doi: 10.1007/s00540-023-03206-3. PMID: 37311899 PMC10263381

[38] Ding XY Zhang MH Liu J Wu D . Recent advances in postoperative delirium in elderly patients: Pathophysiological mechanisms, risk prediction, and therapeutic strategies. Front Neurosci. (2026) 20:1759910. doi: 10.3389/fnins.2026.1759910. PMID: 41725849 PMC12920445

[39] Bellelli G Brathwaite JS Mazzola P . Delirium: A marker of vulnerability in older people. Front Aging Neurosci. (2021) 13:626127. doi: 10.3389/fnagi.2021.626127. PMID: 33994990 PMC8119654

[40] Bellelli G Triolo F Ferrara MC Deiner SG Morandi A Cesari M . Delirium and frailty in older adults: Clinical overlap and biological underpinnings. J Intern Med. (2024) 296:382–98. doi: 10.1111/joim.20047. PMID: 39352688

[41] Abi Chebl J Somasundar P Vognar L Kwon S . Review of frailty in geriatric surgical oncology. Scand J Surg. (2025) 114:276–85. doi: 10.1177/14574969241298872. PMID: 39568134

[42] Ormseth CH LaHue SC Oldham MA Josephson SA Whitaker E Douglas VC . Predisposing and precipitating factors associated with delirium: A systematic review. JAMA Netw Open. (2023) 6:e2249950. doi: 10.1001/jamanetworkopen.2022.49950. PMID: 36607634 PMC9856673

[43] Damluji AA Bernacki G Afilalo J Lyubarova R Orkaby AR Kwak MJ . TAVR in older adults: Moving toward a comprehensive geriatric assessment and away from chronological age: JACC family series. JACC Adv. (2024) 3. 10.1016/j.jacadv.2024.100877 PMC11062620 38694996

[44] Miranda F Gonzalez F Plana MN Zamora J Quinn TJ Seron P . Confusion assessment method for the intensive care unit (CAM-ICU) for the diagnosis of delirium in adults in critical care settings. Cochrane Database Syst Rev. (2023) 11:CD013126. doi: 10.1002/14651858.cd013126.pub2. PMID: 37987526 PMC10661047

[45] Li R Zhang Y Zhu Q Wu Y Song W . The role of anesthesia in peri-operative neurocognitive disorders: Molecular mechanisms and preventive strategies. Fundam Res. (2024) 4:797–805. doi: 10.1016/j.fmre.2023.02.007. PMID: 39161414 PMC11331737

[46] Vacas S Cole DJ Cannesson M . Cognitive decline associated with anesthesia and surgery in older patients. JAMA. (2021). doi: 10.1001/jama.2021.4773. PMID: 34338712 PMC8807795

[47] Jin Z Hu J Ma D . Postoperative delirium: Perioperative assessment, risk reduction, and management. Br J Anaesth. (2020) 125:492–507. doi: 10.1016/j.bja.2020.06.063. PMID: 32798069

[48] Schwesinger A Tsai LT Lang W Mantegazza N Bauernschmitt R Wilhelm MJ . Does comprehensive geriatric assessment reduce the incidence of postoperative delirium? A quasi-experimental study in older adults undergoing transcatheter aortic valve implantation. Clin Interv Aging. (2024) 19:347–55. doi: 10.2147/cia.s448167. PMID: 38434577 PMC10909326

