# Supplementary material for: Association between frailty and postoperative delirium after transcatheter aortic valve replacement: a meta-analysis
Source: Front Psychiatry. 2026 May 21;17:1840158. doi: 10.3389/fpsyt.2026.1840158 (PMC13233520; doi:10.3389/fpsyt.2026.1840158)
Supplement: Supplementary file 1 [file Table1.docx]

**Detailed search strategy for each database**

**PubMed**

**#1** "Frailty"[Mesh] OR frailty[tiab] OR frail[tiab]

**#2** "Transcatheter Aortic Valve Replacement"[Mesh] OR "Transcatheter Aortic Valve Implantation"[tiab] OR TAVI[tiab] OR TAVR[tiab] OR "transcatheter aortic valve replacement"[tiab] OR "transcatheter aortic valve implantation"[tiab]

**#3** "Delirium"[Mesh] OR delirium[tiab] OR confusion[tiab] OR "acute encephalopathy"[tiab] OR "cognitive dysfunction"[tiab] OR "cognitive impairment"[tiab] OR "cognitive disorder"[tiab] OR "altered mental status"[tiab] OR "organic brain syndrome"[tiab]

**#4** #1 AND #2 AND #3

**Embase**

**#1** 'frailty'/exp OR frailty:ti,ab OR frail:ti,ab

**#2** 'transcatheter aortic valve implantation'/exp OR 'transcatheter aortic valve replacement'/exp OR TAVI:ti,ab OR TAVR:ti,ab OR 'transcatheter aortic valve implantation':ti,ab OR 'transcatheter aortic valve replacement':ti,ab

**#3** 'delirium'/exp OR delirium:ti,ab OR confusion:ti,ab OR 'acute encephalopathy':ti,ab OR 'cognitive dysfunction':ti,ab OR 'cognitive impairment':ti,ab OR 'cognitive disorder':ti,ab OR 'altered mental status':ti,ab OR 'organic brain syndrome':ti,ab

**#4** #1 AND #2 AND #3

**Web of Science**

TS = ((frailty OR frail) AND ("transcatheter aortic valve implantation" OR "transcatheter aortic valve replacement" OR TAVI OR TAVR) AND (delirium OR confusion OR "acute encephalopathy" OR "cognitive dysfunction" OR "cognitive impairment" OR "cognitive disorder" OR "altered mental status" OR "organic brain syndrome"))
