# Supplementary material for: Association between frailty and postoperative delirium after transcatheter aortic valve replacement: a meta-analysis
Source: Front Psychiatry. 2026 May 21;17:1840158. doi: 10.3389/fpsyt.2026.1840158 (PMC13233520; doi:10.3389/fpsyt.2026.1840158)

**Supplemental Figure 1** Forest plots for the sensitivity analysis using REML-based methods with Hartung-Knapp adjustment


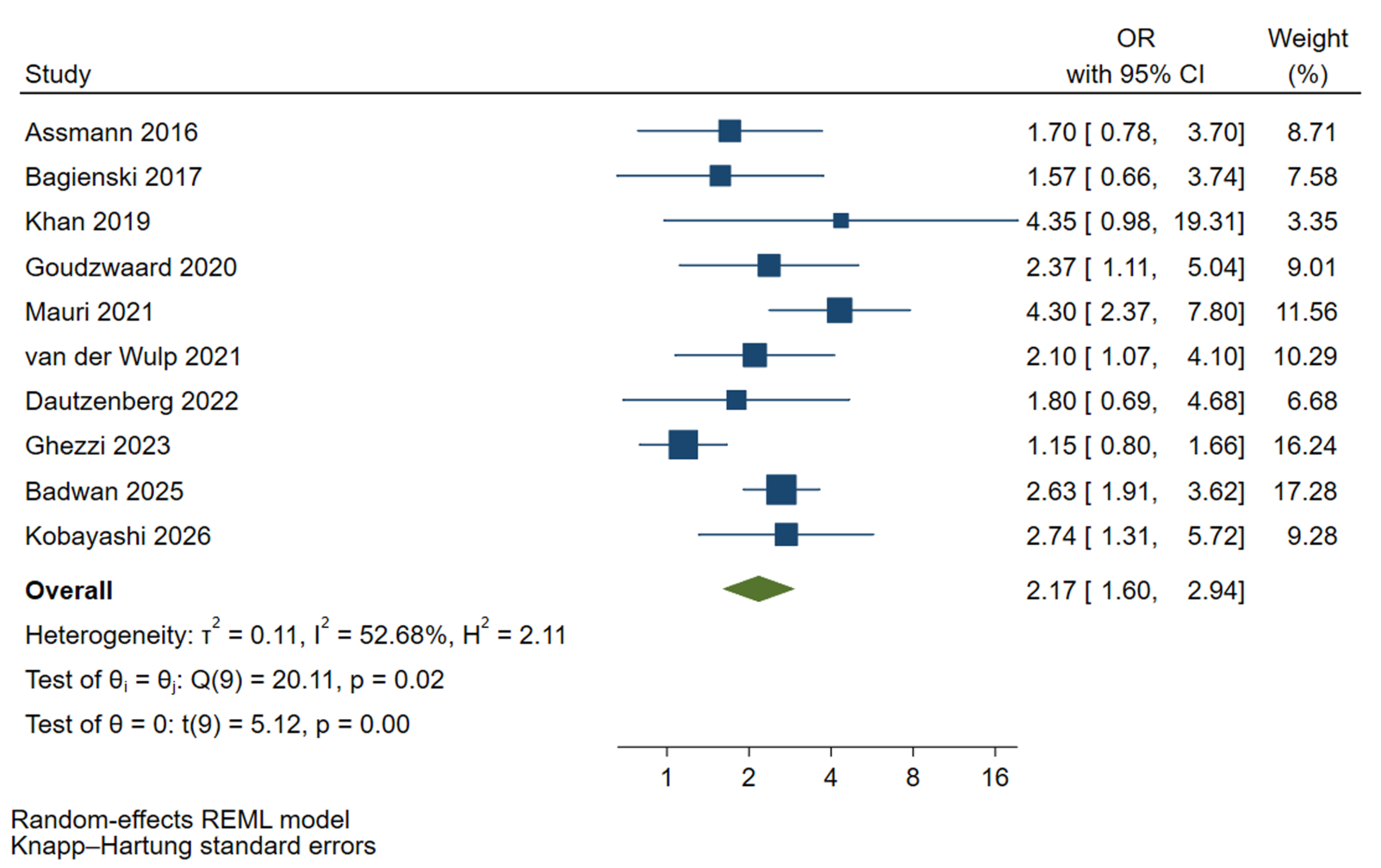

Supplement: Supplementary file 2 [file Table2.docx]
