# Supplementary material for: Association between frailty and postoperative delirium after transcatheter aortic valve replacement: a meta-analysis
Source: Front Psychiatry. 2026 May 21;17:1840158. doi: 10.3389/fpsyt.2026.1840158 (PMC13233520; doi:10.3389/fpsyt.2026.1840158)

Supplemental Figure 2 Forest plots for the sensitivity analysis restricted solely to the studies with most fully adjusted estimates.


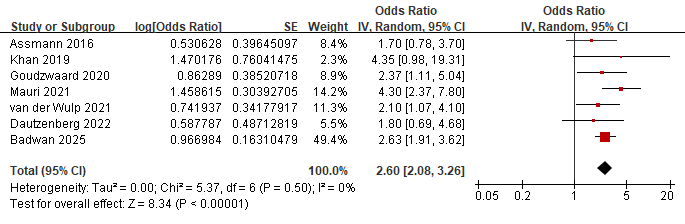

Supplement: Supplementary file 3 [file Table3.docx]
